# Unveiling the Stacking Fault-Driven Phase Transition Delaying Cryogenic Fracture in Fe-Co-Cr-Ni-Mo-C-Based Medium-Entropy Alloy

**DOI:** 10.3390/ma17112502

**Published:** 2024-05-22

**Authors:** Hui Ding, Zhenhang Du, Haifeng Zhang, Yu Liu, Shiteng Zhao, Yonggang Yang, Changjun Wang, Simin Lei, Ruming Geng, Chunxu Wang

**Affiliations:** 1 Central Iron and Steel Research Institute, Beijing 100081, China; 18510913100@163.com (H.D.); zhenhang_du@163.com (Z.D.); 15222628760@163.com (H.Z.); wangchangjun@nercast.com (C.W.); leisiming@nercast.com (S.L.); gengruming@nercast.com (R.G.); 2 School of Materials Science and Engineering, Beihang University, Beijing 100191, China; szhao@buaa.edu.cn; 3 National Engineering Research Center for Advanced Rolling and Intelligent Manufacturing, University of Science and Technology Beijing, Beijing 100083, China; yangyg@ustb.edu.cn

**Keywords:** medium-entropy alloy, cryogenic behavior, twinning, phase transition, stacking fault energy

## Abstract

In this work, the tensile deformation mechanisms of the Fe_55_Co_17.5_Cr_12.5_Ni_10_Mo_5−x_C_x_-based medium-entropy alloy at room temperature (R.T.), 77 K, and 4.2 K are studied. The formation of micro-defects and martensitic transformation to delay the cryogenic fracture are observed. The results show that FeCoCrNiMo_5−x_C_x_-based alloys exhibit outstanding mechanical properties under cryogenic conditions. Under an R.T. condition, the primary contributing mechanism of strain hardening is twinning-induced plasticity (TWIP), whereas at 77 K and 4.2 K, the activation of martensitic transformation-induced plasticity (TRIP) becomes the main strengthening mechanism during cryogenic tensile deformation. Additionally, the carbide precipitation along with increased dislocation density can significantly improve yield and tensile strength. Furthermore, the marked reduction in stacking fault energy (SFE) at cryogenic temperatures can promote mechanisms such as twinning and martensitic transformations, which are pivotal for enhancing ductility under extreme conditions. The Mo_4_C_1_ alloy obtains the optimal strength–ductility combination at cryogenic-to-room temperatures. The tensile strength and elongation of the Mo4C1 alloy are 776 MPa and 50.5% at R.T., 1418 MPa and 71.2% in liquid nitrogen 77 K, 1670 MPa and 80.0% in liquid helium 4.2 K, respectively.

## 1. Introduction

Cryogenic technology plays a crucial role in extreme technologies, such as cryogenic superconductivity, energy transportation, space exploration, etc. Low-temperature applications require high-strength and toughness-matching materials at cryogenic temperatures. These unique performance materials used in low-temperature environments are called low-temperature materials [1]. The face-centered cubic (FCC) structural alloys, such as high-manganese austenitic steel and the Cantor alloy (FeCoCrNiMn alloy), which were invented at the beginning of this century have high low-temperature plasticity and toughness [2,3]. The strength and ductility increase with the decrease in application temperature, providing a novel route to the design of low-temperature structural materials. At present, further improving the strength of the alloy to adapt to the complex and changeable low-temperature application environment has become an active area of research.

In 2004, Ye et al. [4,5] first proposed the concept of a high-entropy alloy (HEA), which is defined as an alloy containing five or more principal elements in equal or near-equal atomic percentages (5–35 at.%). At the same time, Cantor et al. [6] also proposed the design concept of equimolar multi-principal element alloy composition and proposed the FeCoCrNiMn composition alloy. Later, the concept of medium-entropy alloys (MEAs) was introduced; these alloys typically contain 2–4 principal elements with concentrations between 5 and 35 at.%. However, there is no strict distinction between HEAs and MEAs, and alloys with four or more principal elements are often collectively referred to as HEAs in the literature. In this study, the investigated alloys contain four elements (Fe, Co, Cr, Ni) with concentrations above 5 at.% and are therefore referred to as medium-entropy alloys hereafter. In 2013, the low-temperature application potential of high-entropy alloys was discovered, and new design concepts have been continuously explored [7]. Gali and George [8] first showed that the yield strength, tensile strength, and elongation of the FeCoCrNiMn alloy increased simultaneously when the temperature decreased from R.T. to 77 K. Bernd Gludovatz [9] found that the FeCoCrNiMn alloy is an FCC solid solution with excellent comprehensive mechanical properties and exceptional damage resistance. These mechanical properties contradict the conventional inverse relationship between strength and ductility. Otto et al. [10] found that the increased ductility of the FeCoCrNiMn alloy with the decrease in temperature is related to the occurrence of two deformation mechanisms at 77 K, namely dislocation slip and deformation twins. In contrast, only dislocation slip is observed in the alloy deformed at R.T. to explain the high fracture toughness at a cryogenic temperature. Otto et al. [11] attributed the twinning behavior to the low stacking fault energy of the FeCoCrNiMn alloy at cryogenic temperatures, which was about 20~25 mJ m^−2^. Naeem et al. [12] observed the microscopic deformation behavior mechanism of the FeCoCrNiMn alloy at 60% strain using the neutron diffraction method at 15 K and found the activation and synergy of the dislocation slip, stacking fault, twin, and serration, resulting in continuous hardening and uniform extension and showing an excellent tensile strength of ~2.5 GPa and a ductility of 62%. Jo et al. [13] improved the strength of the FeCoCrNiMn alloy by cold rolling; then, partial recrystallization and twins were retained after transient annealing. At 77 K, the yield strength can arrive at 1.0 GPa, and the elongation of 46% can be maintained. In the FeCoCrNiMn alloy, the low-temperature tensile properties are mainly caused by deformation twinning. However, the twinning is difficult to form at R.T. because the decomposed shear stress is lower than the critical twinning stress [14,15]. He et al. [16,17] studied the phase transformation and deformation mechanism of a CoCrNi alloy under tensile load by in situ neutron diffraction and revealed that FCC to HCP phase transformation occurred during the deformation at 15 K. Moon et al. [18] developed a dual-phase Fe_55_Co_17.5_Cr_12.5_Ni_10_Mo_5_ medium-entropy alloy, which has excellent strength and plasticity and outstanding cold work-hardening ability in ultra-low-temperature environments and is related to the activation of stacking faults, deformation twins, and deformation-induced martensitic transformation mechanisms. The alloy undergoes discontinuous plastic deformation (serrated flow) at 0.5~4.2 K. The serrated flow behavior [19,20] is related to the combined effects of dislocation slip, local stress concentration, and martensitic transformation. Bae et al. [21] found that the Fe_55_Co_17.5_Cr_12.5_Ni_10_Mo_5_ alloy exhibits a perfect combination of strength and ductility at cryogenic temperatures. The alloy is composed of an FCC matrix and μ phase precipitates. Mo can act as a strong cemented carbide element in the alloy [22], and the precipitates play an essential role in its strengthening mechanism.

To enhance the performance of alloys, scholars have introduced various strengthening strategies, such as precipitation strengthening, interstitial solid solution strengthening, grain refinement [23], TWIP [24] (twinning-induced plasticity) strengthening, TRIP [25] (transformation-induced plasticity) strengthening, and heterostructure. The purpose of this study is to systematically investigate the deformation mechanisms and mechanical properties of Fe-Co-Cr-Ni-Mo-C-based medium-entropy alloys at different temperatures, with a focus on exploring the influence of carbon content on the low-temperature performance of the alloys. The aim is to provide an in-depth understanding of the deformation behavior and toughening mechanisms of the alloys in environments of extremely low temperatures, providing theoretical guidance for the development of high-performance, low-temperature structural materials.

## 2. Experimental Procedure

**Figure 1:**
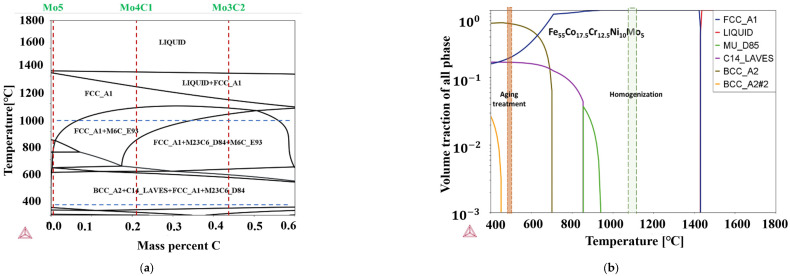

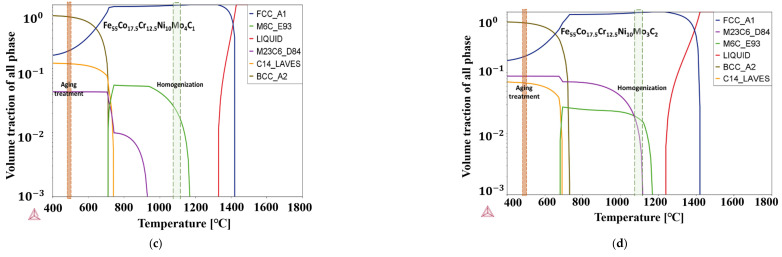
Thermodynamic calculation of Fe_55_Co_17.5_Cr_12.5_Ni_10_Mo_5−x_C_x_ alloys: (**a**) Effect of carbon addition on matrix and carbide precipitation; (**b**) equilibrium phase diagram of Mo_5_ alloy; (**c**) equilibrium phase diagram of Mo_4_C_1_; (**d**) equilibrium phase diagram of Mo_3_C_2._

This work designed the alloy composition based on the prototype alloy composition of Fe_55_Co_17.5_Cr_12.5_Ni_10_Mo_5_ [14]. The carbon element was adopted for solid strengthening and precipitation strengthening. The alloy composition of Fe_55_Co_17.5_Cr_12.5_Ni_10_Mo_5_, Fe_55_Co_17.5_Cr_12.5_Ni_10_Mo_4_C_1_, Fe_55_Co_17.5_Cr_12.5_Ni_10_Mo_3_C_2_ were selected and are abbreviated as Mo_5_, Mo_4_C_1_, and Mo_3_C_2_, respectively, in the following. The high-purity (>99.9 wt.%) raw materials were prepared using the vacuum induction melting process. The nominal and measured compositions are compared in Table 1; “Bal” stands for “Balance”, indicating that Fe is the remaining component. Afterwards, the thermodynamic calculation using Thermo-Calc 2023a software was performed to primarily determine the types and amounts of carbide precipitation, as shown in Figure 1. The as-cast alloy was forged above 1373 K and cut into tensile and impact blanks, followed by heat treatment. The heat treatment system was determined with the solid solution at 1373 K for 1 h following water cooling and the aging treatment at 753 K for 5 h of water cooling.

The tensile test, impact test, and microstructure observation were carried out after heat treatment. The standard rod-shaped tensile specimen with diameter d = 5 mm and L = 5 d was used for the tensile specimen. XRD, OM, SEM, EDS, EBSD, and TEM were used to observe the microstructure. Tensile tests at R.T. and 77 K were carried out using the Landmark series MTS universal testing machine. The tensile rate during the loading process was set to 2.5 × 10^−4^ S^−1^. The SANS CMT5105 S tensile testing machine was used to test the 4.2 K tensile specimen. A JBN-300B impact testing machine was used for the impact test. XRD was performed using a Brook D8 ADVANCE X-ray diffractometer with a Co target. The tube voltage and current were set to 35 kV and 40 mA, respectively. The scanning speed was 5° min^−1^ with a step size of 0.020°. The 2θ irradiation angle ranged from 45° to 115°. The scanning electron microscope model was an FEI Quanta 650 FEG microscope equipped with an EDS probe. The transmission samples were thinned by a Gatan691 ion thinning instrument and observed by an FEI Talos F200 X transmission electron microscope. EBSD was observed using HKL Symmetry/EDAX Velocity Super model equipment.

## 3. Experimental Results

### 3.1. Mechanical Property of Mo_5_ Alloy

**Figure 2:**
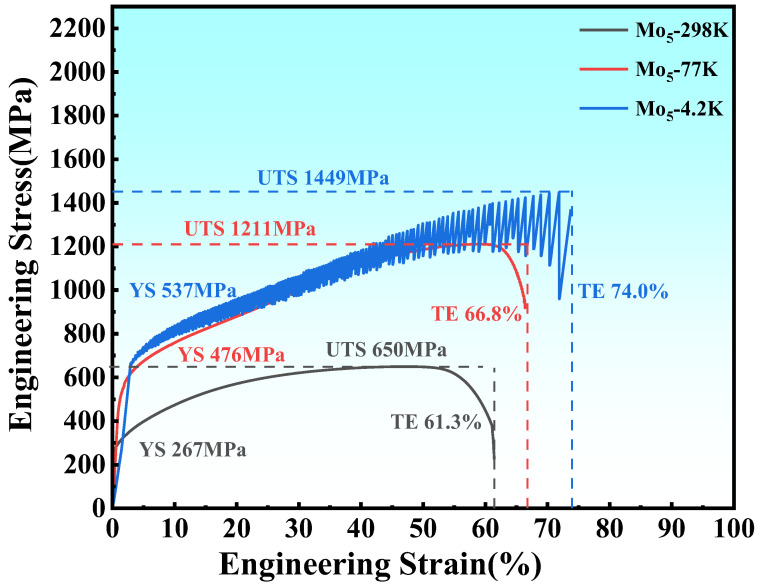
Stress–strain curve of tensile property.

The tensile stress–strain curves of the alloy at R.T., 77 K, and 4.2 K are shown in Figure 2. The yield strength and tensile strength of the Mo_5_ alloy at R.T. are 267 and 650 MPa, respectively, and the elongation after fracture is 61.3%. With the decrease in temperature, the yield strength, tensile strength, and elongation after fracture increase simultaneously. The yield strength and tensile strength of the alloy at 77 K are 476 and 1211 MPa, respectively, and the elongation after fracture is 66.8%. The yield and tensile strength at 4.2 K are 537 and 1449 MPa, respectively, and the fracture elongation is 74.0%. The tensile and impact properties of the alloy are shown in Table 2.

### 3.2. Microstructure Characterization of Mo_5_ Alloy

**Figure 3:**
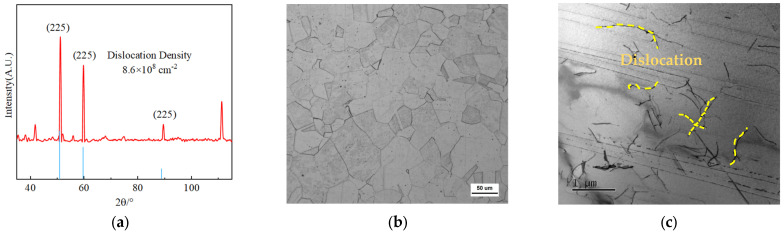

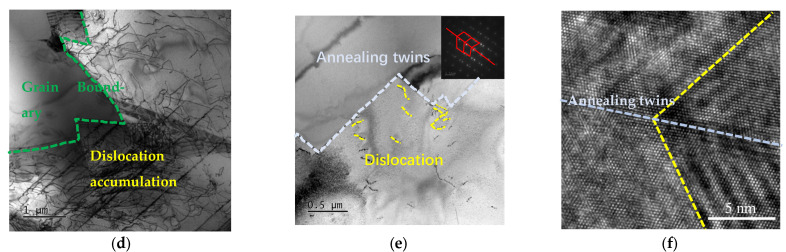
Microstructure characterization and fractography adjacent to the necking region: (**a**) XRD pattern of Mo5 alloy; (**b**) metallographic images of Mo_5_ alloy; (**c**–**e**) micro-defects characterization by TEM analysis; (**f**) high-resolution TEM image to determine twin formation.

The microstructure characterization of the alloy matrix is shown in Figure 3. The XRD results are shown in Figure 3a. The metallographic images of the Mo_5_ alloy are shown in Figure 3b. The alloy comprises equiaxed grains; their size is statistically measured as 36.8 μm. Moreover, there are distributed amounts of annealing twins inside the grains. After performing the tensile test at 77 K, the TEM images of the Mo_5_ alloy to characterize the micro-defects are shown in Figure 3c–e. As shown in Figure 3d,e, large amounts of dislocation accumulate near the boundaries of the grain and annealing twins, which is determined by the diffraction spot. Furthermore, the high-resolution image of annealing twins is shown in Figure 3f, and the lattice arrangement of the annealing twins can be seen.

**Figure 4:**
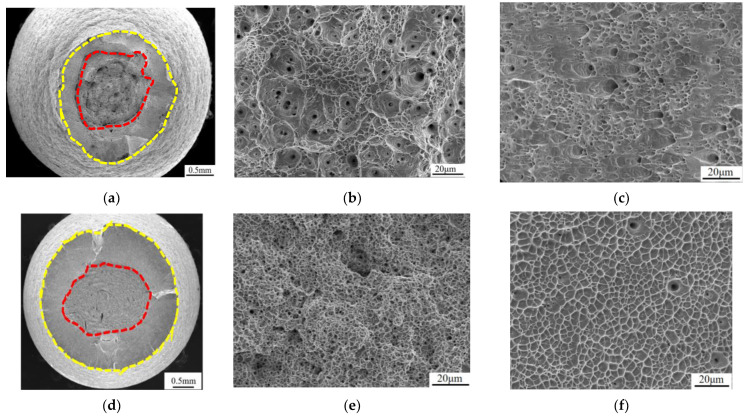

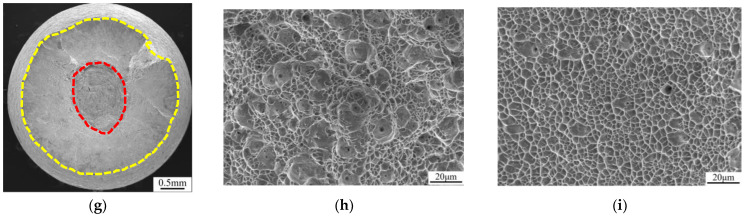
Tensile fracture analysis: R.T. (**a**) fracture overall, (**b**) fiber area morphology, (**c**) shear lip area morphology; 77 K (**d**) the whole fracture surface, (**e**) the fiber morphology, and (**f**) the shear lip morphology; 4.2 K (**g**) fracture overall, (**h**) fiber area morphology, (**i**) shear lip area morphology.

The tensile fracture morphology of the tested alloy at R.T., 77 K, and 4.2 K is shown in Figure 4. The tensile fracture necking of the test alloy at R.T. is obvious, and the fracture surface is mainly composed of a fiber area and shear lip area. In the observation, it can be found that the fiber area and shear lip area are equivalent. The fracture fiber area is composed of large and deep dimples. The dimples in the shear lip area of the fracture are not noticeable, and the dimples adhere to each other and are torn. The 77 K tensile fracture also has obvious necking, and the fiber area is densely distributed with small dimples. It is observed that the dimple has a certain depth and that the shear lip area has extensive and uniform dimples. The necking of the tensile fracture at 4.2 K is obvious, and there are two kinds of dimples in the fiber area. The morphology of the shear lip zone is consistent with the fracture morphology after 77 K tensile. After analysis, the tensile fracture morphology of the alloy at three temperatures is slightly different, but it is undeniable that the alloy has excellent toughness at three temperatures. As the temperature decreases, the shrinkage rate of the alloy fracture decreases, which may be caused by the increase in the alloy strength.

### 3.3. Effect of Carbon Addition on Mechanical Property and Microstructure

**Figure 5:**
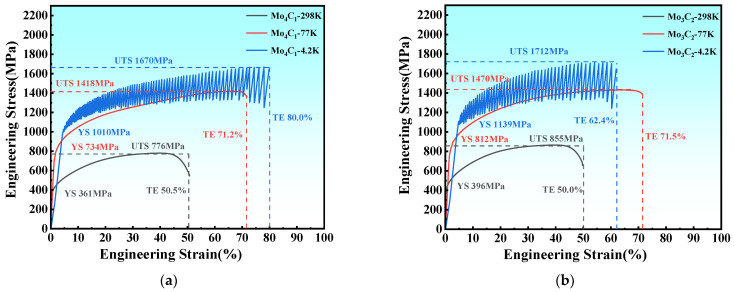
Engineering stress–plastic curve of tensile property at R.T., 77 K, and 4.2 K: (**a**) Mo_4_C_1_; (**b**) Mo_3_C_2_.

**Figure 6:**
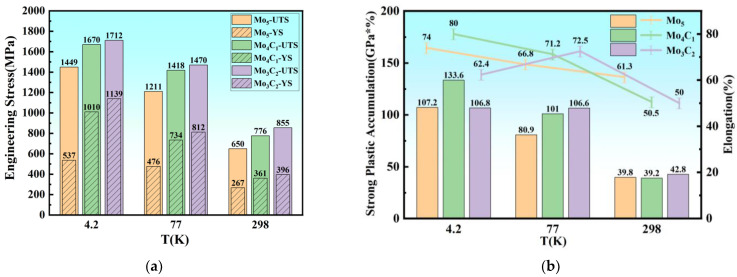
Comparison of mechanical properties of Mo_5_, Mo_4_C_1_, and Mo_3_C_2_: (**a**) ultimate tensile stress and yield stress; (**b**) product of strength and elongation.

The tensile stress–strain curves of the Mo_4_C_1_ and Mo_3_C_2_ alloys at R.T., 77 K, and 4.2 K are shown in Figure 5. The yield stress, ultimate tensile stress, elongation, and the product of the strength and elongation of Fe_55_Co_17.5_Cr_12.5_Ni_10_Mo_5−x_C_x_ at various temperatures are compared in Figure 6. At R.T., the tensile strength and yield strength of the Mo_4_C_1_ alloy are 776 MPa and 361 MPa, respectively. Compared with tensile properties at R.T., the tensile strength and yield strength under liquid nitrogen are 1418 and 734 MPa, respectively, increasing by 82.79% and 103.6%. Under liquid helium, the tensile strength and yield strength are 1670 and 1010 MPa, rising by 115.3% and 180.2%. The products of strength and elongation under liquid nitrogen and liquid helium are 101.0 and 133.6 GPa%, respectively. At R.T., the tensile strength and yield strength of the Mo_3_C_2_ alloy are 855 MPa and 396 MPa, respectively. Under liquid nitrogen, the tensile and yield strengths are 1470 and 812 MPa, which are increases of 71.93% and 105.05%, respectively. Under liquid helium, the tensile strength and yield strength are 1712 and 1139 MPa, which are increases of 106.1% and 187.6%, respectively. The products of strength and elongation under liquid nitrogen and liquid helium are 106.6 and 106.8 GPa%, respectively. The addition of carbon significantly improves the strength of the alloys at each temperature, and the strengthening effect is enhanced dramatically with the decrease in temperature.

**Figure 7:**
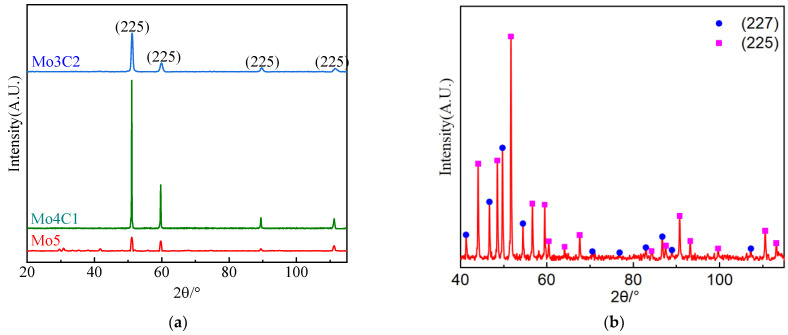
XRD analysis spectrum of the Fe_55_Co_17.5_Cr_12.5_Ni_10_Mo_5−x_C_x_ alloys: (**a**) matrix; (**b**) carbides.

**Figure 8:**
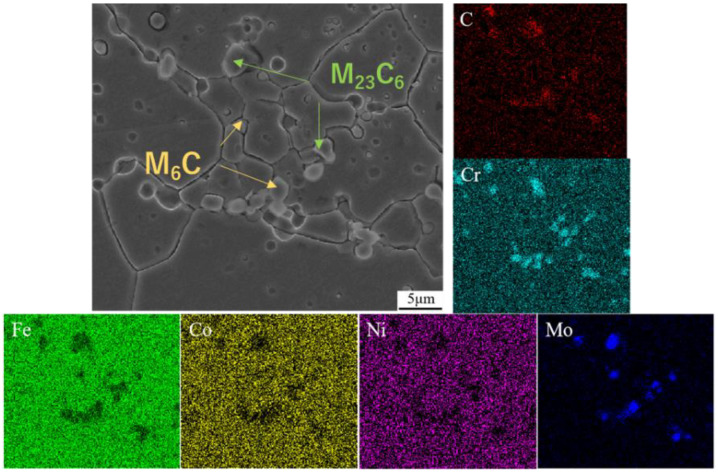
Scanning morphology and element analysis of matrix and carbides in Fe_55_Co_17.5_Cr_12.5_Ni_10_Mo_5−x_C_x_ alloys.

The XRD pattern of the Fe_55_Co_17.5_Cr_12.5_Ni_10_Mo_5−x_C_x_ alloys is shown in Figure 7. After calibrating diffraction peaks, it is found that the matrix of the alloys is an FCC structure, and there exists a trace amount of carbides. Due to the small carbide quantity (less than ≤1%), it is necessary to carry out XRD qualitative phase analysis after extraction by electrolysis, as shown in Figure 7b. The carbide types are primarily determined as M_6_C and M_23_C_6_ carbides, respectively. To further determine the carbide types, the SEM-EDS is employed to observe the carbides’ morphology and analyze the element distribution, as shown in Figure 8. It can be observed that a distribution of Cr, Mo, and C positive elements and a distribution of Fe, Co, and Ni negative elements exist and that the amounts of carbides precipitate on the grain boundary. Based on the Cr, Mo, and C element segregation types, the chemical composition of the M_6_C and M_23_C_6_ carbides can be identified as Mo_3_Co_3_C and Cr_21_Mo_2_C_6_, respectively. Furthermore, the M_23_C_6_ carbide occupies the main part as the Cr-rich area is mainly distributed. Moreover, the dislocation density of the alloys was measured, as listed in Table 3. As can be seen, with the increase in carbon content, the dislocation density exponentially ascends.

**Figure 9:**
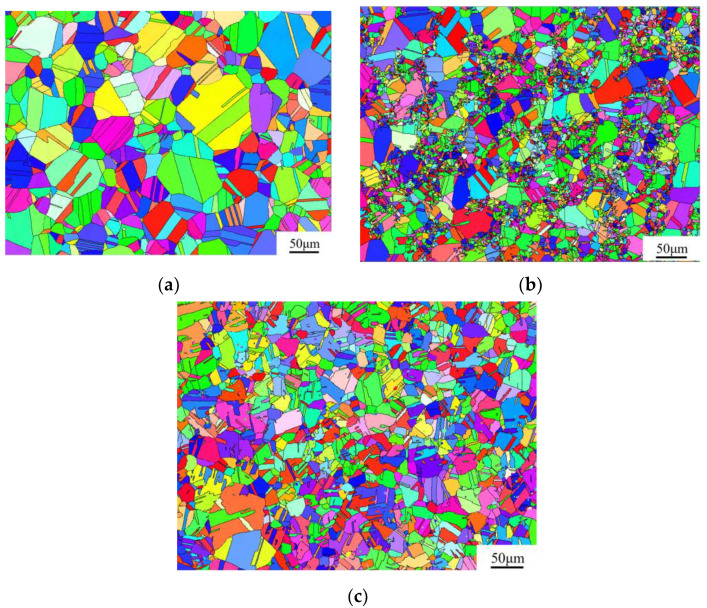
EBSD analysis of Fe_55_Co_17.5_Cr_12.5_Ni_10_Mo_5−x_C_x_ alloys: (**a**) Mo_5_ alloy; (**b**) Mo_4_C_1_ alloy; and (**c**) Mo_3_C_2_ alloy.

The EBSD analysis of Fe_55_Co_17.5_Cr_12.5_Ni_10_Mo_5−x_C_x_ alloys is shown in Figure 9. The results show that vast amounts of annealing twins distribute inside the grain. The grain size range of the Mo_5_ alloy is in the range of 1.8 and 105.6 μm, and the average grain size is 36.8 μm. Nevertheless, as the carbon content increases, the grain of the Mo_4_C_1_ alloy apparently downsizes. The grain size range of the Mo_4_C_1_ alloy is in the range of 1.8 to 54.2 μm, and the average grain size is 17.0 μm. The forging treatment of the Mo_4_C_1_ and Mo_3_C_2_ alloys with various carbon contents affects the grain breakup to a more significant degree. That is to say, the higher the carbon content is, the more evident the effect is. For the Mo_3_C_2_ alloy, the grain size range and average grain size are 0.7 ~ 23.6 μm and 8.0 μm. Furthermore, the grain size distribution of the Mo_4_C_1_ alloy is uneven since the grain boundary is broken during the forging process, and the grains gradually enlarge during the recrystallization process. At the same time, the dense and dispersed carbide precipitation hinders the further growth of grain boundaries to form the finer grains. In contrast, the M_6_C and M_23_C_6_ carbides grow up and aggregate for the Mo_3_C_2_ alloy in a relatively higher carbon content—the lower nucleation amount results in far fewer grains during recrystallization than the former.

**Figure 10:**
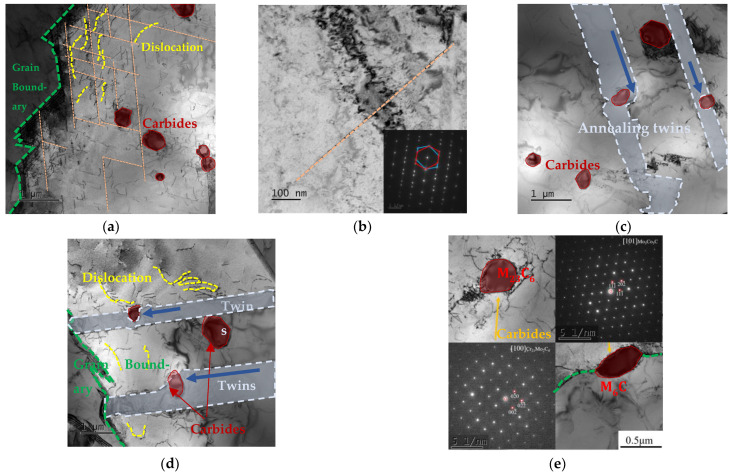
TEM analysis, diffraction patterns of Fe_55_Co_17.5_Cr_12.5_Ni_10_Mo_5−x_C_x_ alloys: (**a**,**d**) TEM analysis of Mo_4_C_1_ and Mo_3_C_2_ alloys; (**b**) deformation nano-twins determined by diffraction patterns; (**c**) annealing twins and carbides in Mo_4_C_1_ alloy; (**e**) diffraction patterns of carbides.

The microstructure morphology and carbide precipitation of the Fe_55_Co_17.5_Cr_12.5_Ni_10_Mo_5−x_C_x_ alloys are studied by using TEM characterization, as shown in Figure 10. Figure 10a shows that large amounts of dislocations and slip bands randomly distribute inside the grains of the Mo_4_C_1_ alloy. The grain boundary hinders the proliferation of dislocations, leading to dislocation accumulation near the grain boundary into the dislocation network. Additionally, the deformation nano-twin formation is determined by diffraction patterns in Figure 10b. The annealing twins are across the grains, and the stacking fault densely forms along the grain boundary. The carbide size is at the level of 300~500 µm, and for the interaction between microscopic defects, the carbides hinder the slip of dislocations and the growth of annealing twins, as shown in Figure 10c. The TEM micrography of the Mo_3_C_2_ alloy is shown in Figure 10d,e. The size of the precipitated carbides enlarges to the level of 450~800 µm. The grain boundary and annealing twins seriously hinder the increment of dislocations to form the crowded dislocation networks near the boundary. Furthermore, as mentioned above in Table 3, the dislocation density exponentially ascends with increased carbon content. The carbides can be determined as M6C and M23C6 types by calibrating the diffraction spot, as shown in Figure 10e. The dislocation entanglement and pile-up can be observed near the carbides; in addition, the growth paths of the annealing twins bypass the carbides, which may be the reason for the increase in strength along with the addition of carbon content. The carbides on the grain and the twins’ boundaries are generally larger, which can be inferred from the fact that the nucleation and growth are more likely in the micro-defects position.

### 3.4. Phase Transition during Cryogenic Tensile Deformation

**Figure 11:**
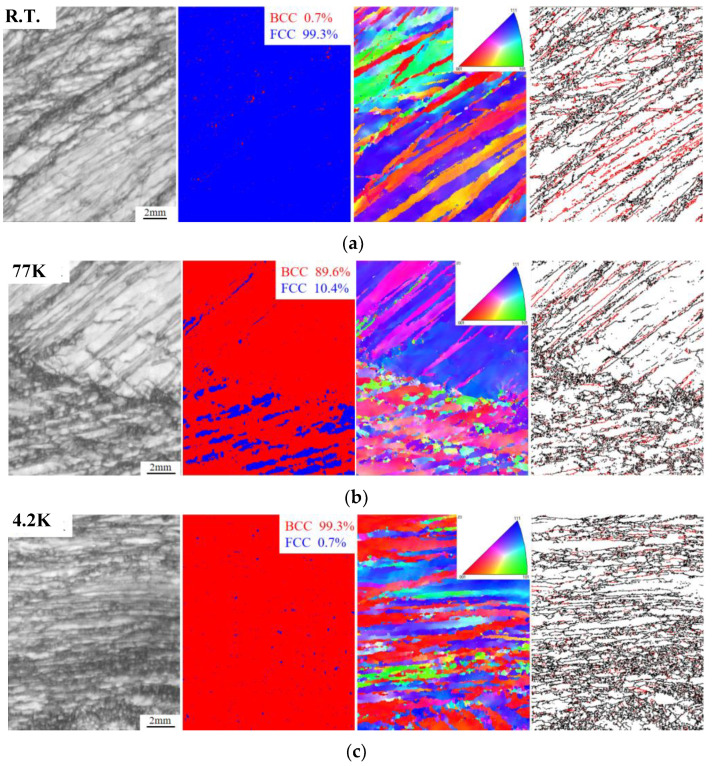
EBSD images of tensile fracture at R.T., 77 K, 4.2 K for Mo_5_ alloy: (**a**) B.C. diagram, phase diagram, IPF diagram, and twin distribution of necking region at R.T; (**b**) B.C. diagram, phase diagram, IPF diagram, and twin distribution of the necking region at 77 K; (**c**) B.C. diagram, phase diagram, IPF diagram, and twin distribution of necking region at 4.2 K. (Red marks are the twin boundary.)

The tensile tests of the alloy were carried out at R.T., 77 K, and 4.2 K, and the necking area of the fracture was analyzed by EBSD characterization. As shown in Figure 11, it can be found that there is no apparent martensitic transformation in the necking area of the tensile fracture at ambient temperature. The grains with this orientation have a large amount of deformation during the tensile process, which promotes the activation of the phase transformation mechanism. The distribution diagram has large amounts of twins, and the deformation at R.T. triggers large amounts of twinning behaviors. Under this condition, the martensitic transformation mechanism has not been activated. The primary contributing mechanism of strain hardening in tensile engineering is large numbers of twinning behaviors. For the necking area adjacent to the tensile fracture carried out at 77 K, the BCC martensitic transformation occurs at a greater volume fraction magnification of 89.6%. The grains along the (111) orientation in the IPF diagram have been almost completely transformed into the BCC phase, while the grains along the (001) orientation are not entirely transformed. It can be found that the density of twining has declined substantially in the distribution map. At the cryogenic temperature of 4.2 K, the martensitic transformation has entirely occurred in the grains along the (001) orientation, and a further reduction in twinning formation can be seen in the distribution map.

**Figure 12:**
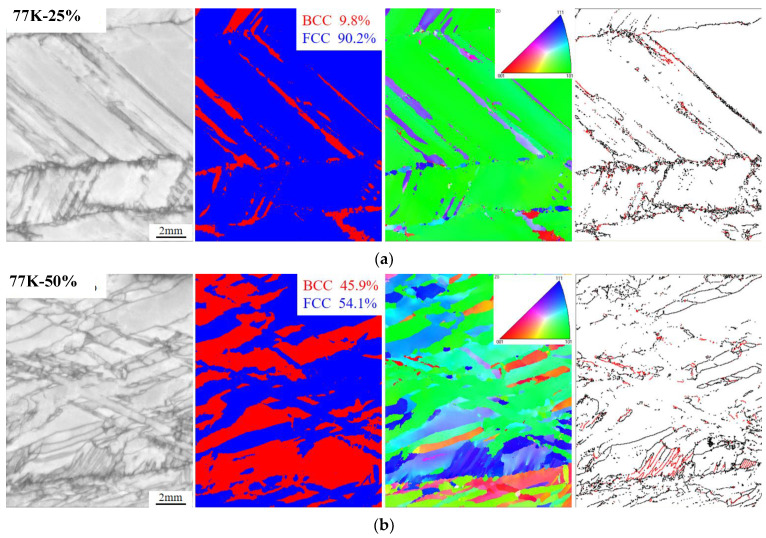
EBSD images of different tensile strain necking regions at 77 K: (**a**) B.C. diagram, phase distribution diagram, IPF diagram, and twin distribution of necking area of Mo_5_ alloy at 77 K tensile 25% deformation; (**b**) B.C. diagram, phase distribution diagram, IPF diagram, and twin distribution of necking area of Mo_5_ alloy at 77 K tensile 50% deformation. (Red marks are the twin boundary.)

After the experimental alloy was stretched to 25% and 50% deformation at 77 K, EBSD analysis was performed on the necking area, as shown in Figure 12. The purpose of this test is to compare with the necking area after tensile fracture at 77 K, and to explore the deformation-induced twinning behavior and the stage of deformation-induced martensitic transformation. It can be seen that the FCC phase content in the tensile necking region of the 25% deformation alloy is 90.2%, which still accounts for the main part. The deformation-induced martensitic transformation began at 25% deformation, with a small amount of twin distribution. The content of the FCC phase in the tensile necking region of the 50% deformation alloy is 54.1%; that is, nearly half of the FCC phase in the matrix has undergone a martensitic transformation under the current deformation, and the number of twins has increased compared with the 25% deformation. As shown in Figure 12b, when the deformation is 66%, that is, when the tensile specimen is broken, 89.6% of the matrix undergoes a martensitic transformation, and the number of deformation-induced martensitic transformations increases, and the number of twins also increases. The current experiment proves that the alloy’s deformation-induced twinning behavior and deformation-induced martensitic transformation during the tensile process occur in the work hardening stage. With the work hardening, the proportion of twinning behavior and martensitic transformation in the matrix increases.

## 4. Discussion

In this study, we delved into the enhanced ductility and strength of the FeCoCrNiMo_5−x_C_x_-based medium-entropy alloy at cryogenic temperatures. Moreover, we conducted an in-depth analysis of the impact of carbides on the properties of the alloy. For the Mo_5_ and Mo_4_C_1_ alloys, yield strength and tensile strength showed a notable improvement when the temperatures were reduced to 77 K and 4.2 K. This improvement can be associated with the increased critical shear stress for plastic deformation and the resistance from the dislocation interactions under cryogenic conditions. It is clearly shown that the yield strength at cryogenic temperatures significantly surpasses the yield strength at ambient temperature. The results reveal that at reduced temperatures, deformation-induced twinning and martensitic transformation mechanisms are activated, significantly enhancing the work-hardening effects observable in the tensile engineering stress–strain curves. The dynamic Hall–Petch effect, which is attributable to twinning during deformation, is believed to be the underlying reason for the observed strain-hardening mechanics in the alloy.

Regarding the high-entropy alloy (HEA), stacking fault energy (SFE) is regarded as one critical intrinsic material parameter that influences mechanical properties, plastic deformation behavior, and the characteristics and mechanism of phase transformation. Especially at a cryogenic temperature, the alteration of the SFE is particularly instrumental in dictating the activation of distinct deformation mechanisms, particularly stacking fault, twinning, and martensitic transformation.

**Figure 13:**
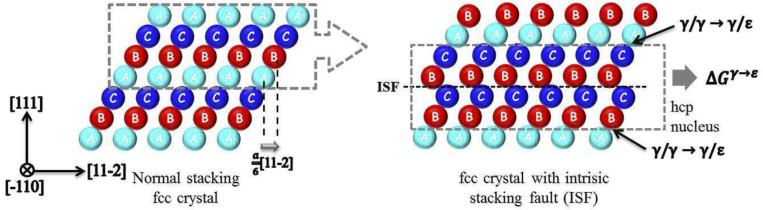
Stacking fault energy calculation diagram [17].

To address the temperature dependency of SFE, we adopted a combined approach that integrated computational thermodynamics with first-principles calculations and further enforced this framework with the quasi-harmonic approximation (QHA) [26]. This methodology allows an in-depth insight into the microscopic deformation mechanisms, guided by the overarching results from macroscopic mechanical testing. The calculation of SFE is built upon the thermodynamic model proposed by Olson and Cohen, which equates the γ-fcc to ε-hcp phase transition to the extraction of a thin ε-hcp phase layer within an γ-fcc crystal structure [27], as shown in Figure 13. The calculation formula for stacking fault energy is as follows:

(1) $$\gamma_{ISF}=2\rho ∆G^{\gamma \to \varepsilon }+2\sigma^{\gamma /\varepsilon }$$

where $\gamma_{ISF}$ is the intrinsic stacking fault energy (mJ/m^2^), $∆G^{\gamma \to \varepsilon }$ is the Gibbs free energy of the γ-fcc to ε-hcp phase transformation, $\sigma^{\gamma /\varepsilon }$ is the interfacial energy between γ and ε phases, and ρ is the geometric factor.

**Figure 14:**
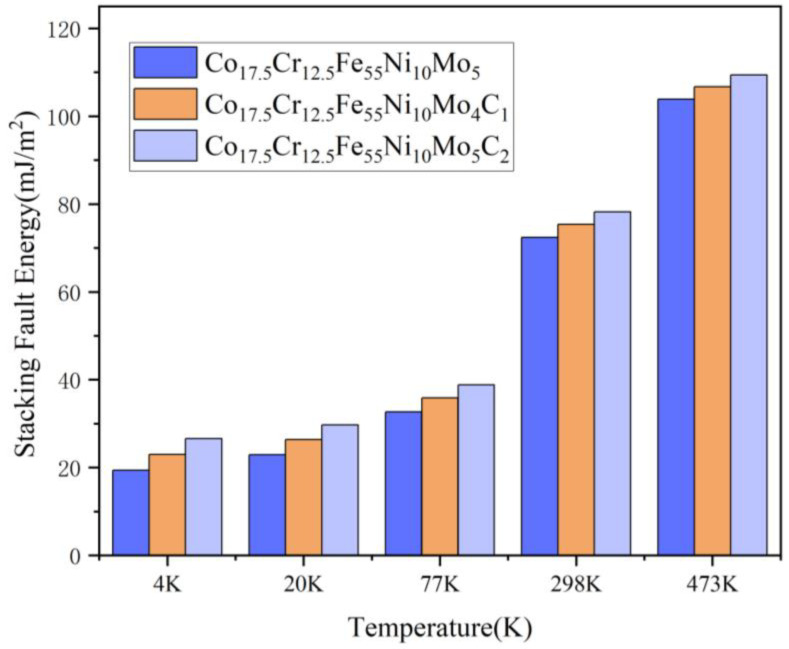
Stacking fault energy calculation.

The computational results indicate a significant decrement in SFE at the cryogenic temperatures of 77 K and 4.2 K. More precisely, for the Mo_5_ alloy, we determined the SFE to be 72.1 mJ/m^2^ at R.T., decreasing to 32.33 mJ/m^2^ at 77 K, and further dropping to 18.97 mJ/m^2^ at 4.2 K. The marked reduction in SFE at cryogenic temperatures can promote mechanisms such as twinning and martensitic transformations, which are pivotal for enhancing ductility under extreme conditions. Additionally, the carbon addition augments the stacking fault energy to a certain extent, as shown in Figure 14. The impact of SFE on the mechanical properties due to carbon addition on solid strengthening and ductility reduction can be negligible. The main effect of carbon addition in decisively determining the cryogenic property is the strengthening of precipitation.

To reveal the interaction mechanism between the twins, phase transformation, and the carbides, for the Mo_5_ alloy, the formation of twins (TWIP) and phase transformation (TRIP), because of the SFE reduction along with the temperature decline, is the leading reason for delaying the fracture. For the Mo_4_C_1_ alloy, in the same manner, the micro-defect formation significantly impacts the improvement of plasticity, and the main effect of the precipitated carbides is to improve the strength. For the Mo_3_C_2_ alloy, the fracture plasticity plummets as the temperature declines to 4.2 K, identifying why the carbides, namely M₆C and M₂₃C₆, impede dislocation slip and twin growth. With the augmenting of the carbon content, the dislocation density significantly increases, providing additional effects of solid solution strengthening on the enhancement of the strength. Notably, at cryogenic temperatures, although twinning formation due to reduced SFE can improve the ductility, the precipitation of carbides suppressed twinning and thus severely deteriorated fracture elongation. Additionally, for the tensile testing conducted under the 4.2 K condition, the alloy exhibited stress and strain collapse phenomena during plastic deformation, known as serrated flow behavior, which indicates that the microstructure defects, even the ductile-to-brittle transition, form and interact in real time during the tensile deformation process. Nevertheless, as the carbon content ascends, the ability to delay fracture due to micro-defect formation weakens during the cryogenic tensile testing, resulting in the reduction in ductility and the product of strength and elongation. Overall, the combined effects of decreased SFE, increased twinning, and martensitic transformations at cryogenic temperatures, along with the strengthening influence of carbides, contribute to the superior mechanical properties of FeCoCrNiMo_5−x_C_x_-based medium-entropy alloys at cryogenic temperatures.

## 5. Conclusions

This research systematically studies the Fe-Co-Cr-Ni-Mo-C-based medium-entropy alloy’s mechanical properties and microstructure at R.T., 77 K, and 4.2 K. The mechanisms of micro-defect activation during deformation under cryogenic conditions are the main concern. The main findings can be summarized as follows:

1. FeCoCrNiMo_5−x_C_x_-based alloys exhibit outstanding mechanical properties under cryogenic conditions. Particularly for the Mo_4_C_1_ alloy, the strength and ductility increase simultaneously from R.T. to 4.2 K. The tensile strength and elongation of the Mo_4_C_1_ alloy are 776 MPa and 50.5% at R.T., 1418 MPa and 71.2% in liquid nitrogen 77 K, and 1670 MPa and 80.0% in liquid helium 4.2 K, respectively.
2. Under the R.T. condition, the primary contributing mechanism of strain hardening is twinning-induced plasticity. At 77 K and 4.2 K, the activation of martensitic transformation to partially replace the twining mechanism (TRIP) became the main reason for plasticity improvement during cryogenic tensile deformation.
3. The precipitated carbides, namely M₆C and M₂₃C₆, along with increased dislocation density can significantly improve yield and tensile strength. As the temperature freezes to 4.2 K, the plasticity of the Mo_3_C_2_ alloy deteriorates, identifying the reason why the carbides hinder the dislocation slip and twin growth.
4. The significant decrease in stacking fault energy (SFE) observed at cryogenic temperatures can facilitate processes like twinning and martensitic transformations, which play a crucial role in improving ductility under extreme conditions. The addition of carbon increases the stacking fault energy to some degree. However, the primary impact of carbon addition on the determination of cryogenic properties remains the reinforcement of precipitation mechanisms.

## Figures and Tables

**Table 1:** The composition of Fe_55_Co_17.5_Cr_12.5_Ni_10_Mo_5−x_C_x_ medium-entropy alloys.

Alloy	Co-wt%	Cr-wt%	Mo-wt%	Ni-wt%	C-wt%	H- wt%	N-wt%	O-wt%	Fe-wt%
Mo_5_—Nominal Composition	17.72	11.17	8.24	10.08					Bal.
Mo_5_—Measured composition	17.80	11.0	8.25	10.12	0.014	0.00008	0.0020	0.0058	Bal.
Mo_4_C_1_—Nominal Composition	17.98	11.33	6.69	10.23	0.21				Bal.
Mo_4_C_1_—Measured composition	18.09	11.26	6.72	10.27	0.21	0.00010	0.0016	0.0025	Bal.
Mo_3_C_2_—Nominal Composition	18.25	11.50	5.09	10.38	0.42				Bal.
Mo_3_C_2_—Measured composition	18.27	11.45	5.05	10.41	0.44	0.00010	0.0015	0.0014	Bal.

**Table 2:** Tensile and impact properties of Mo_5_ alloy.

Alloys	Temperature (K)	R_m_ (MPa)	R_p0.2_ (MPa)	A (%)	Z (%)
Mo_5_	R.T.	650	267	61.3	83.5
	77 K	1211	476	66.8	74.0
	4.2 K	1449	537	74.0	64.0

Rm: Ultimate tensile strength; Rp0.2: Yield strength at 0.2% offset; A: Percentage elongation after fracture; Z: Percentage reduction in area.

**Table 3:** Dislocation density of Fe_55_Co_17.5_Cr_12.5_Ni_10_Mo_5−x_C_x_ alloys.

Alloys	Mo_5_	Mo_4_C_1_	Mo_3_C_2_
Dislocation density (cm^−2^)	8.60 × 10^8^	1.82 × 10^9^	3.97 × 10^11^

## Data Availability

Data are contained within the article.
